# Transformative Learning: Visiting a Multigenerational Living Community in Switzerland

**DOI:** 10.1093/geroni/igaf122.4034

**Published:** 2025-12-31

**Authors:** Jennifer Leszczynski, T Caitlin Vasquez-O’Brien

**Affiliations:** Eastern Connecticut State University, Willimantic, Connecticut, United States; Eastern Connecticut State University, Willimantic, Connecticut, United States

## Abstract

Global studies are transformative for students (Jean-Francois, Sharma, & Obi, 2023) and intergenerational experiences help students to view aging through a cross-cultural lens and better understand the importance of intergenerational connections (Montepare & Leedahl, 2020) and aging in place. In May 2025, 26 undergraduates and two faculty members visited The Foundry (Giesserei) – a grassroots community designed specifically for multigenerational living in Winterthur, Switzerland. Including 155 apartments and housing individuals across the lifespan, the community contains a library, preschool, restaurant and art gallery. As the first Americans to tour the facility, we will highlight our perspectives of this unique opportunity.

